# Effects of 10 KM run on foot morphology and bilateral symmetry in male recreational runners

**DOI:** 10.3389/fbioe.2024.1427418

**Published:** 2024-08-13

**Authors:** Shiwei Mo, Feifei Lu, Chuhao Li, Huan Zhao

**Affiliations:** ^1^ Laboratory of Human Kinesiology and Performance, School of Physical Education, Shenzhen University, Shenzhen, Guangdong, China; ^2^ School of Fashion and Textiles, Hong Kong Polytechnic University, Kowloon, Hong Kong SAR, China

**Keywords:** distance running, foot arch, arch stiffness index, 3D scan, symmetry index

## Abstract

Foot morphology and arch integrity do not remain constant during a running bout. Previous studies have reported inconsistent changes in foot sizes and arch parameters and this discrepancy may be related to the variation in their test duration, e.g., 15-min treadmill run vs. 30 KM trial. Hence, this study sought to evaluate the change in foot morphology, arch integrity and bilateral symmetry after a 10 KM run among 19 male recreational runners. Before and after the run, a portable foot scanner was used to capture the 3-dimensional foot images and measure foot dimensions in both weight-bearing and non-weight-bearing conditions. Foot arch integrity was quantified by arch height ratio, arch height index, and arch stiffness index (ASI). Bilateral symmetry was evaluated by calculating the symmetry index. Increased foot length (*p* = 0.007; $\eta_{p}^{2}$ = 0.18) and decreased ball girth (*p* = 0.038; $\eta_{p}^{2}$ = 0.11) were demonstrated following the run with absolute differences of less than 2 mm. Navicular height, dorsum height, arch height ratio and arch height index significantly decreased after the run (*p* < 0.001; $\eta_{p}^{2}$ ≥0.30) whereas ASI increased (*p* < 0.001, $\eta_{p}^{2}$ = 0.33) and navicular height drop reduced (*p* < 0.001, $\eta_{p}^{2}$ = 0.37). Significances of symmetry index were only demonstrated for navicular height (*p* = 0.019, effect size = 0.37) and arch height ratio (*p* = 0.019, effect size = 0.42). A few changes in foot morphology were detected but a reduction in foot arch height was demonstrated, which may give shoe manufacturers insights into shoe design. Male recreational runners were recommended to choose shoes with arch support for maintaining foot arch function during a 10 KM run.

## 1 Introduction

Footwear is one of the most important gears for runners. Footwear designers and manufacturers usually highlight the footwear function such as shock attenuation, arch support, carbon fiber plate and lightweight. Nevertheless, footwear fit is always the primary concern among runners. Ill-fitting footwear would cause poor comfort perception, which would decrease running performance and increase the risk of injuries such as blistering, chafing, black toes, bunions and pain. Footwear fit is attained through matching the shape of footwear and foot, which indicates that footwear should be designed to accommodate an individual’s foot. However, foot morphology continuously varied during running. Mei et al. (2018) reported decreased foot volume and ball width whereas Boni et al. (2012) demonstrated no alterations in foot volume and Shiotani et al. (2020) reported no changes in foot length after a 10 KM run. Theoretically, foot morphology would become enlarged because running would increase the blood flow in foot muscles, produce the peripheral vasodilatation of foot superficial tissues and elevate in-shoe temperature. Such inconsistent changes in foot morphology between the aforementioned studies and theory bring greater challenges for footwear designers or runners in determining the right footwear. 10 KM run is consistently considered among the most popular running distances and with the greatest number of competitions. It has attracted the largest number of participations regardless of sex, age and running experience. Beginners often considered it the next milestone after a 5 KM race and marathon runners would benchmark their training progress by competing a 10 KM race and predict their finishing time of the forthcoming marathon. Considering the inconclusive opinions on the change of foot morphology and the popularity of 10 KM run, further studies are therefore urgently required to clarify how feet adapt to the 10 KM run.

Besides foot morphology, the medial longitudinal foot arch, as a unique function structure of the foot, plays a key role in shock attenuation during running. The arched shape allows the foot to work like a spring. Any alterations of foot arch would cause harm to the body tissues and affect running biomechanics (Butler et al., 2006; Powell et al., 2011; Williams et al., 2014; Woźniacka et al., 2019), such as malalignment of lower-limb joints and abnormal distribution of feet loads, thereby increasing the risk of injuries such as plantar fasciitis and stress fracture of the metatarsal bones. Distance running was reported to alter the foot arch in both shape and stiffness. Female joggers, in comparison to their non-exercise counterparts, were found to have a higher frequency of flattened foot arch (Maslon et al., 2017) whereas recreational marathon runners showed higher foot arch than non-habitual exercisers (Chen et al., 2022). Acute effects were also investigated and a collapsed foot arch (flattened foot arch) was demonstrated immediately after a long-distance run (i.e., 20 km, half marathon, 35 km, full marathon) (Cowley and Marsden, 2013; Fukano and Iso, 2016; Mei et al., 2018; Fukano et al., 2021). A flattened foot arch (flatfoot) is usually accompanied with malalignment of foot (i.e., hindfoot valgus, forefoot abduction) and knee joint (i.e., knee valgus), which would alter distribution of joint loads during running. Regarding the 10 KM run, the findings are conflicting. Mei et al. (Mei et al., 2018) detected an unchanged foot arch height whereas Shiotani et al. (Shiotani et al., 2020) found reduced foot arch height immediately after running 10 km. Shiotani et al. (Shiotani et al., 2020) also reported significantly decreased arch stiffness after a 10 KM run. Another study (Boyer et al., 2014) reported no changes in both foot arch shape and stiffness after a 45-min run at a comfortable pace (the distance is close to 10 km). It remains inconclusive regarding the effects of a 10 KM run on the foot arch.

Runners with similar foot morphology between left and right feet displayed asymmetrical running biomechanics (Mo et al., 2020), which would result in unequal load distribution, thereby increasing the odds of injury on the side with a higher load (Zifchock et al., 2006; Zifchock et al., 2008). On the other hand, running may worsen symmetrical foot morphology due to the asymmetrical running biomechanics. Regular female joggers were found to show more asymmetrical loads in the metatarsal bones compared with their non-jogger counterparts (Maslon et al., 2017); recreational marathon runners instead of non-habitual exercisers displayed bilateral differences in foot morphology (Chen et al., 2022). Asymmetrical changes were also demonstrated immediately after distance running. Foot posture index was reported to be different between right and left feet after a half marathon (Cowley and Marsden, 2013); acute asymmetrical changes were detected on the foot arch after a full marathon (Fukano et al., 2021). To date, no studies have investigated foot asymmetry in recreational runners after a 10 KM run.

Different running biomechanics were demonstrated between male and female (Xie et al., 2022); foot morphology and structure showed significant sex-specific differences (Hong et al., 2011; Fukano and Fukubayashi, 2012); and sex was not differentiated (Boyer et al., 2014; Mei et al., 2018; Shiotani et al., 2020) or only female runners were recruited (Maslon et al., 2017) when investigating the change of foot structure following a long-distance running in previous studies. Thus, this study primarily investigated the acute effects of a 10 KM run on foot morphology and foot arch structure in male recreational runners. It was hypothesized that foot morphology would be enlarged, foot arch height would be decreased, and foot arch stiffness would be increased after the run. Our secondary purpose was to explore whether the aforementioned changes between right and left feet are symmetrical or not and we hypothesized that foot asymmetry would be significantly increased after the run.

## 2 Materials and methods

### 2.1 Participants

Nineteen healthy male recreational runners voluntarily participated in this study. Their mean ± standard deviation (SD) age, height, and body weight were respectively 23.0 ± 6.6 years, 1.76 ± 0.05 m, and 66.0 ± 6.1 kg. Their running experience and minimal weekly running volume were respectively 4.2 ± 4.4 years and 41.6 ± 24.8 km, and their personal best performance of the 10 KM run was 0:47:24 (hours: minutes: seconds). Before data collection, all the runners signed an informed consent statement preapproved by the University Medical Ethics Committee (No. PN-2021–009).

According to the reported differences of foot morphology measurements (i.e., foot length) and foot arch parameters (i.e., arch stiffness index) before and after running 10 km in our previous study (Mo et al., 2023), an *a priori* power analysis suggested that at least 16 participants would be sufficient to reach the expected effect size using alpha = 0.05 and beta = 0.20.

### 2.2 Equipment

A portable hand-held three-dimensional (3D) scanner along with the Shining software (EinScan Pro 2X Plus, SHINING 3D Tech. Co., Hangzhou, China) were utilized to capture and obtain all the runners’ 3D foot model in this study. The hand-held scanner consists of a central projector and two side monochrome cameras which are located at the extremity of the instrument. During operation, the scanner is gripped by the operator and moved around the object of interest, meantime perforated scanned point clouds are generated and turned into a mesh, thereby a perforated surface is produced and successfully modeled the object of interest in the Shining software. The 3D scanner allows a maximum scanning speed of 30 frames/sec (or 1.5 M points/sec) and a single slice volume of up to 312 × 204 mm with a volume accuracy of 0.3 mm/m. The reported scanning accuracy was up to 0.1 mm, which is much less than the standard accuracy requirement of 2 mm (International Organization for Standardization, 2010). Because of its small and portable size, the 3D scanner has been largely used to capture anthropometric dimensions of the foot in previous studies (Liu et al., 2019; Zhang et al., 2022; Susanto et al., 2023; Zhang et al., 2023).

### 2.3 Data collection

All runners were asked to complete a 10 KM run on a 400-m outdoor synthetic rubber track at their fastest pace in the early morning in November (21°C–25°C). To minimize varied effects resulting from different shoe models, the same model of running shoes (City Run, ANTA Sports Products Ltd., China) was assigned to all runners during the run. The running shoes are one kind of traditional running shoes with the following characteristics: foam midsole of E-TPU material, heel-to-toe drop of 15 mm and stack height at the heel of 28 mm (EUR 41), and without any carbon plate or arch support. Such model of running shoes was chosen because of the popularity among the recreational runners in China and the similarity to the running shoe model of the recruited runners. More importantly, the running shoes do not provide any extra arch support or protection to foot arch during running. Before the 10 KM run, they were given enough time to find the right shoe size (length and width) with the best perception of shoe-fit through trying on shoes of different sizes. All runners were required not to do any strenuous exercises 24 h before to avoid fatigue accumulation. Before and immediately after the run, a senior technician scanned bilateral feet for each runner in both standing and seated postures using the 3D scanner under environmental light conditions. The scan was conducted in a fitness room next to the outdoor track with indoor temperature (20°C–22°C) and humidity (50%–60%) being controlled constant. Specifically, the runner was initially asked to stand statically on a mirror plate with feet shoulder-width apart to ensure evenly distributing body weight on bilateral feet; thereafter he was required to sit in a standard posture (back resting on the chair, 90 degrees of knee flexion, neutral ankle position) with feet placed weightlessly on the mirror plate; the technician gripped the instrument and rotated it around the runner’s feet with slow and uniform movements, meantime the scanning quality was checked to ensure all 3D triangulated meshes of the feet being produced through the proprietary Shining software on the notebook. It took less than 90 s for each scan, and the runner was asked to maintain a stationary position without any foot motion throughout the scan and thereafter to rest and adjust body posture for the remaining scan. The whole scanning procedure (standing posture, rest and seated posture) took less than 5 min. The scanning data files were saved as object files (.obj) for post-processing of foot anthropometric dimensions.

### 2.4 Data processing and analysis

**FIGURE 1:**
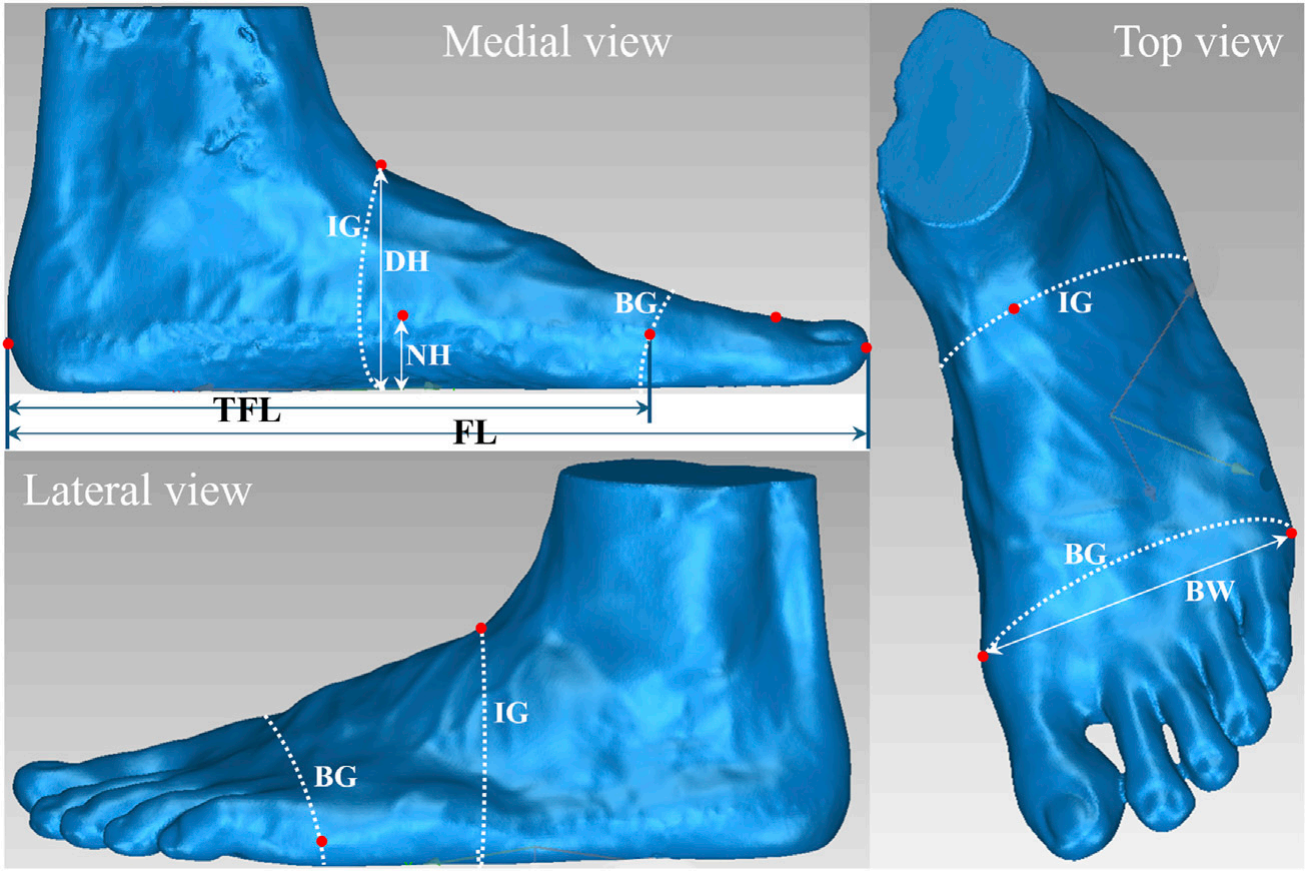
Definition of the eight foot anthropometric parameters: foot length (FL); truncated foot length (TFL), ball width (BW), hallux height (HH), navicular height (NH), dorsum height (DH), ball girth (BG) and instep girth (IG).

In this study, the 3D foot model files of each runner were processed using the Geomagic software (Geomagic^®^ Design X™, 3D SYSTEM, United States) and eight foot morphology measurements were taken from the 3D foot model by an experienced research assistant based on a previously established protocol (Williams and McClay, 2000; Witana et al., 2006; Price and Nester, 2016). Measurement reliabilities of the research assistant were confirmed with mean absolute errors of less than 2.1 mm and the intra-rater reliability (intraclass correlation coefficients) of greater than 0.88. The eight measurements (Figure 1) could be classified into four categories and their definitions are listed as following.

• Length: foot length was defined as the distance from the most posterior portion of the calcaneus to the end of the longest toe; and truncated foot length was defined as the distance from the most posterior portion of the calcaneus to the center of first metatarsal joint.
• Width: ball width was the distance between the first and fifth metatarsal joints.
• Height: hallux height was defined as the vertical height from the floor to the highest point of the hallux; navicular height was defined as the vertical height of the most anterior-inferior portion of the navicular bone from the floor; and dorsum height was defined as the vertical height of the highest point on dorsum at the position of 50% of the foot length from heel.
• Girth: ball girth was defined as the perimeter of the first and fifth metatarsal joints; and instep girth was the circumference passing through dorsal junction of the foot and leg.

Another four advanced parameters were also computed to quantify the shape and stiffness of the foot arch structure: arch height ratio, arch height index, navicular height drop, arch stiffness index (ASI). Arch height ratio was defined as the foot length normalized navicular height and was presented in percentage. Arch height index was the ratio of the dorsum height and truncated foot length, which eliminates the effect of toe length, and the value close to zero indicates a more flattened foot arch (Williams and McClay, 2000; Xu et al., 2019). Navicular height drop was the absolute difference of the navicular height between seated (weightless) and standing postures (around 50% body weight). ASI was defined as the ratio of navicular height in standing and seated postures. Both navicular height drop and ASI denotes the deformation capability (or stiffness) of foot arch structure. A higher ASI value indicates a stiffer foot arch structure, which means that the foot arch is less likely to be deformed under external load (Xu et al., 2019).

Finally, based on the eight foot morphology measurements and four foot arch parameters of bilateral feet, symmetric index (SI) was calculated using the following equation:

$${SI}_{X}=\frac{\left|X_{L}-X_{R}\right|}{\left(X_{L}+X_{R}\right)\times 0.5}\times 100$$

where *X* represents a specific parameter, *L* is left and *R* is right. In general, SI = 0 indicates absolute symmetrical changes between left and right feet, whereas a larger SI value means more asymmetrical changes (Mo et al., 2020).

### 2.5 Statistical analysis

All statistical analyses were performed using SPSS 26.0 for Windows (SPSS, IBM, Chicago, United States) and the level of significance was set at 0.05. Mean and SD were presented for each dependent variable. The distribution of all the variables was checked using the Shapiro-Wilk test. Normal distribution was confirmed for all the foot morphology and arch structure measurements (*p* > 0.05) and two-way (bilateral feet × 2 time points) repeated measures analysis of variance (ANOVA) was used for the comparison between foot/time points. Partial eta squared ( $\eta_{p}^{2}$ ) was calculated to quantify the effect size, where values of 0.01, 0.06 and 0.14 respectively represent small, moderate and large effects (Cohen, 2013). In addition, non-normal distributions were confirmed for most of the SI variables by the Shapiro-Wilk test (*p* > 0.05). The Wilcoxon two-sample paired signed-rank tests were therefore used to determine whether the SI variables were significantly different before and after the run. The effect size was calculated as *z* statistic divided by the square root of the sample size, where effects were interpreted as negligible (<0.10), small (<0.30), moderate (<0.50) and large (≥0.50) (Williams and McClay, 2000).

## 3 Results

### 3.1 Foot morphology measurements

Results of the foot morphology measurements before and after the 10 KM run are presented in Table 1. There were no interaction effects and foot effects. Significant time effects were only detected in foot length (*p* = 0.007) and ball girth (*p* = 0.038) with effect size ( $\eta_{p}^{2}$ ) of 0.18 and 0.11, respectively. Overall, foot length became longer and ball girth was smaller after the run. However, the absolute change was relatively trivial (less than 2.0 mm).

**TABLE 1:** Comparison of foot anthropometric measurements in standing posture before and after the 10 km run (Mean ± Standard deviation).

	Before		After		Two-way ANOVA		
	Right	Left	Right	Left	Interaction effect	Time effect	Foot effect
Foot length (mm)	258.5 ± 9.4	259.5 ± 8.3	259.2 ± 9.0	259.9 ± 8.6	F = 0.29 *p* = 0.59 $\eta_{p}^{2}$ = 0.01	F = 8.29 *p* = **0.007** $\eta_{p}^{2}$ = 0.18	F = 0.09 *p* = 0.77 $\eta_{p}^{2}$ = 0.002
Truncated foot length (mm)	183.8 ± 8.7	188.5 ± 7.2	184.7 ± 9.4	189.1 ± 8.2	F = 0.18 *p* = 0.68 $\eta_{p}^{2}$ = 0.01	F = 3.15 *p* = 0.08 $\eta_{p}^{2}$ = 0.08	F = 2.99 *p* = 0.09 $\eta_{p}^{2}$ = 0.07
Ball width (mm)	102.2 ± 3.7	103.4 ± 3.7	102.5 ± 3.7	103.6 ± 3.7	F = 0.19 *p* = 0.66 $\eta_{p}^{2}$ = 0.01	F = 1.42 *p* = 0.24 $\eta_{p}^{2}$ = 0.04	F = 0.96 *p* = 0.34 $\eta_{p}^{2}$ = 0.03
Ball girth (mm)	249.2 ± 8.9	251.1 ± 6.9	248.4 ± 8.6	249.5 ± 7.7	F = 0.40 *p* = 0.53 $\eta_{p}^{2}$ = 0.01	F = 4.61 *p* = **0.038** $\eta_{p}^{2}$ = 0.11	F = 0.36 *p* = 0.55 $\eta_{p}^{2}$ = 0.01
Instep girth (mm)	326.0 ± 11.6	326.3 ± 11.4	327.4 ± 12.0	326.1 ± 12.2	F = 1.62 *p* = 0.21 $\eta_{p}^{2}$ = 0.04	F = 0.88 *p* = 0.36 $\eta_{p}^{2}$ = 0.02	F = 0.02 *p* = 0.88 $\eta_{p}^{2}$ <0.01
Hallux height (mm)	23.8 ± 1.8	23.4 ± 2.0	23.7 ± 1.7	23.3 ± 1.8	F = 0.01 *p* = 0.91 $\eta_{p}^{2}$ <0.01	F = 0.60 *p* = 0.45 $\eta_{p}^{2}$ = 0.02	F = 0.45 *p* = 0.51 $\eta_{p}^{2}$ = 0.01

### 3.2 Foot arch structure measurements

All measurements quantifying the shape and stiffness of the foot arch structure are displayed in Table 2. There were no interaction effects and foot effects. Time effects were observed for all the foot arch measurements (*p* < 0.001) and the effect sizes were large (0.31 ≤ $\eta_{p}^{2}$ ≤ 037). In general, ASI significantly increased whereas other foot arch measurements (navicular height, dorsum height, arch height ratio, arch height index and navicular height drop) significantly decreased after the run.

**TABLE 2:** Comparison of foot arch structure measurements in standing posture and foot arch function variables before and after the 10 km run (Mean ± Standard deviation).

	Before		After		Two-way ANOVA		
	Right	Left	Right	Left	Interaction effect	Time effect	Foot effect
Navicular height (mm)	21.9 ± 4.3	21.4 ± 5.3	20.3 ± 3.7	20.0 ± 4.4	F = 0.09 *p* = 0.76 $\eta_{p}^{2}$ <0.01	F = 20.40 *p* < **0.001** $\eta_{p}^{2}$ = 0.35	F = 0.08 *p* = 0.78 $\eta_{p}^{2}$ <0.01
Dorsum height (mm)	67.5 ± 4.3	66.5 ± 4.8	66.5 ± 4.3	65.1 ± 5.1	F = 0.45 *p* = 0.51 $\eta_{p}^{2}$ = 0.01	F = 16.59 *p* < **0.001** $\eta_{p}^{2}$ = 0.30	F = 0.65 *p* = 0.43 $\eta_{p}^{2}$ = 0.02
Arch height ratio (%)	8.49 ± 1.75	8.26 ± 2.09	7.85 ± 1.45	7.73 ± 1.74	F = 0.18 *p* = 0.68 $\eta_{p}^{2}$ = 0.01	F = 20.59 *p* < **0.001** $\eta_{p}^{2}$ = 0.35	F = 0.10 *p* = 0.75 $\eta_{p}^{2}$ <0.01
Arch height index	0.368 ± 0.027	0.353 ± 0.026	0.360 ± 0.026	0.345 ± 0.028	F = 0.31 *p* = 0.58 $\eta_{p}^{2}$ = 0.01	F = 17.41 *p* < **0.001** $\eta_{p}^{2}$ = 0.31	F = 3.59 *p* = 0.07 $\eta_{p}^{2}$ = 0.09
Navicular height drop (mm)	4.6 ± 2.5	4.3 ± 1.8	3.0 ± 2.2	3.0 ± 1.6	F = 0.23 *p* = 0.64 $\eta_{p}^{2}$ = 0.01	F = 22.58 *p* < **0.001** $\eta_{p}^{2}$ = 0.37	F = 0.13 *p* = 0.72 $\eta_{p}^{2}$ = 0.01
Arch stiffness index	0.817 ± 0.086	0.825 ± 0.073	0.882 ± 0.086	0.876 ± 0.070	F = 0.32 *p* = 0.57 $\eta_{p}^{2}$ = 0.01	F = 18.54 *p* < **0.001** $\eta_{p}^{2}$ = 0.33	F = 0.01 *p* = 0.93 $\eta_{p}^{2}$ <0.01

### 3.3 Bilateral symmetry

**FIGURE 2:**
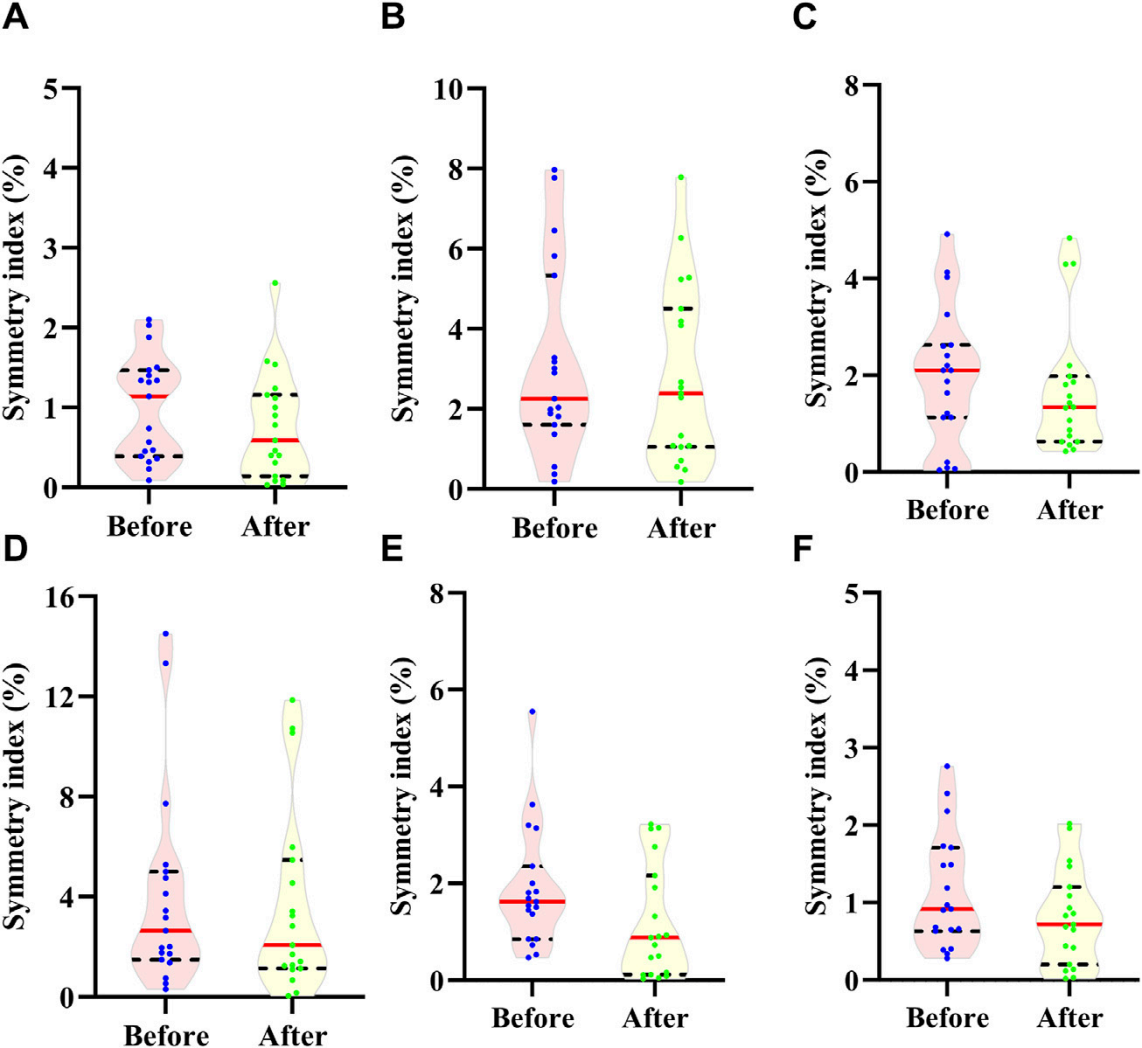
Comparison of symmetry index for **(A)** foot length, **(B)** truncated foot length, **(C)** ball width, **(D)** hallux height, **(E)** ball girth and **(F)** instep girth before and after the 10 km run. Dot indicates the value of each participant; Red line indicates median; Dash line indices quartile.

Regarding the foot morphology, SI values for all the selected measurements were compared before and after the run in Figure 2. Overall, the SI values were relatively small (median of the group <3%) whereas the within group variances were relatively large for the truncated foot length (Figure 2B) and the hallux height (Figure 2D). SI for all the foot morphology measurements were not significantly different before and after the run (*p* > 0.05).

**FIGURE 3:**
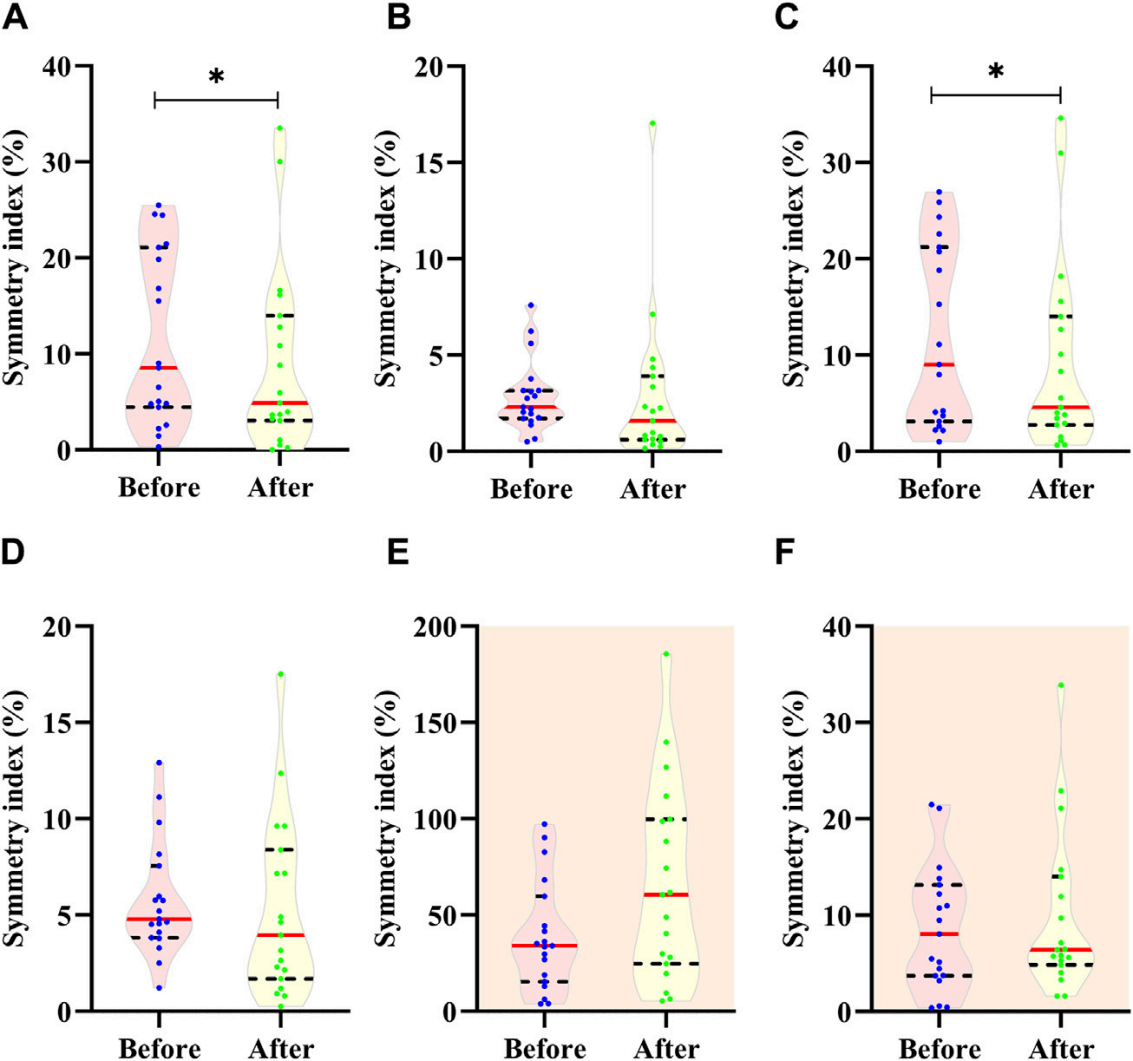
Comparison of symmetry index for **(A)** navicular height, **(B)** dorsum height, **(C)** arch height ration, **(D)** arch height index, **(E)** navicular height drop and **(F)** arch stiffness index before and after the 10 km run. Dot indicates the value of each participant; Red line indicates median; Dash line indices quartile.

Regarding the foot arch structure, SI values for all the measurements were displayed in Figure 3. SI values varied greatly from 1.6% (median for the dorsum height after the run; Figure 3B) to 60.6% (median for the navicular height drop after the run; Figure 3E). SI values for each measurement also showed great variances among runners, e.g., the minimum value was 5.4% while the maximum value reached 185.6% for the navicular height drop after the run (Figure 3E). Statistically, significant differences before and after the run were detected only for the navicular height (*p* = 0.019, effect size = 0.37; Figure 3A) and arch height ratio (*p* = 0.019, effect size = 0.42; Figure 3C).

## 4 Discussion

This study demonstrated significant increase in foot length and decrease in ball girth after the 10 KM run. Such findings were partially consistent with our original hypothesis. Swells were reported in healthy populations after the strenuous cardiovascular exercises due to increased blood flow in exercising muscles (McWhorter et al., 2003) and the weight bearing physical activities like running were reported to cause foot and ankle swelling (Cloughley and Mawdsley, 1995). In our study, the increment of foot length after the 10 KM run may be partially attributed to the swells of the forefoot resulted by the increased blood flow in the superficial tissues and foot muscles. Additionally, the in-shoe temperature and foot superficial temperature were previously found to increase significantly after a distance running (Gil-Calvo et al., 2019; Jimenez-Perez et al., 2020), which, according to the theory of Thermal Expansion, may also contribute to the increment in our study. Two previous studies (Mei et al., 2018; Shiotani et al., 2020), which were the only two studies compared foot morphology before and after a 10 KM run, reported inconsistent findings. Mei et al. (2018) reported significantly decreased ball width and unchanged foot length, truncated foot length and ball girth in recreational runners; Shiotani et al. (2020) only measured foot length and demonstrated no change in both runners and untrained men after the run. The recruited participants with different running experience (varied running experience vs recreational runners or untrained men), different road surfaces (synthetic rubber track vs. asphalt road), shoe condition (a pair of traditional running shoes with the same shoe model vs. runners’ own shoes), 3D foot measurement tool (Einscan Pro 2X Plus SHINING 3D vs. Easy-Foot-Scan OrthoBaltic, JMS-2100CU Dream GP) and even different environmental temperature may together contribute to the inconsistency between our study and the aforementioned two studies. However, no matter the increment in our study or the decrement reported by Mei et al. (2018), the absolute differences were less than 2.0 mm. On the other hand, feet were reported to shrink after the 10 KM run and the loss of bodily fluids through heavy sweating (dehydration) was considered the contributor (Mei et al., 2018). This study was conducted on outdoor track in the early morning in November. The apparent temperature (21°C–25°C) was pleasant and no runners reported heavy sweating after the run. Standing on this fact, we reserved our opinions that the 10 KM run would significantly alter male recreational runners’ foot morphology from a particular dimension. Such small alterations may not lead to ill-fitting issues as there is sufficient in-shoe space between feet and shoes. Reviewing previous related studies, foot volume was tend to increase after a short run (i.e., 15 min or less than 5 km) (Cloughley and Mawdsley, 1995) and decrease following a long-distance run (i.e., 20 km, half marathon, 35 km, or marathon) (Fukano and Iso, 2016; Mei et al., 2018). It sounds that 10 km is the turning point of running distance that inducing opposite changes of foot morphology. Yet, a solid experimental study is required to confirm such assumption.

Regarding the foot arch structure, this study supported our original hypothesis and demonstrated significant alterations for all the selected foot arch measurements after the 10 KM run. Specifically, the navicular height, dorsum height, arch height ration and arch height index significantly decreased, indicating that the foot arch collapsed after the run. Similarly, Shiotani et al. (2020) reported decreased navicular height and arch height ratio after a 10 KM run. Such deformation of the foot arch was also observed after running a longer distance such as 20 km, 35 km, marathon (Fukano and Iso, 2016; Mei et al., 2018; Fukano et al., 2021). The foot arch largely relies on joints, muscles and soft tissues (i.e., plantar fascia) for maintaining its half dome shape. Besides absorbing landing impact, the plantar fascia is repeatedly stretched and recoiled for gaining and releasing elastic energy during running, which would accumulate the neuromuscular fatigue (Fiolkowski et al., 2003; Headlee et al., 2008; O’Connor and Hamill, 2004) and alter its mechanical property (Cheung et al., 2004; Cowley and Marsden, 2013), thereby failing to maintain the initial shape. Meantime, ASI significantly increased and navicular height drop significantly reduced after the run. The two measurements are used to assess stiffness/flexibility of foot arch because both quantify changes of foot arch height during transition from non-weightbearing to weightbearing state. Our findings indicated that the foot arch structure became stiffer (less deformation) after the run. However, contrary to our findings, Shiotani et al. (2020) found that the navicular height drop increased after 10 KM run in both runners and untrained men, indicating reduced foot arch stiffness, and they blamed it to the decreased stiffness of the plantar fascia at the proximal site. Collapsed foot arch shape, altered mechanical property of ligaments and tendons and fatigued foot muscles together contributed to the stiffer foot arch structure observed in this study. Firstly, the collapsed foot arch shape after the run would narrow the gap of the small joints between tarsal and metatarsal bones, thereby tightening bone structure and restricting their movement. Furthermore, the ligaments and tendons were stretched when foot arch became lower. Foot length significantly increased after the run in the current study, which may stretch the plantar fascia, linking the two supporting ends (metatarsal heads and calcaneus) of the foot arch. The stretched plantar fascia would be less flexible when comparing to its natural state. Finally, the fatigued foot muscles after the run may also contribute the increased foot arch stiffness as fatigue would increase muscle stiffness. However, there are lacking direct data in this study. Such explanations are required to be further investigated in the future.

Gait asymmetry is another factor tightly associated with running-related injuries. Although foot morphology symmetrically changed, SI for the navicular height and arch height ratio was significantly reduced after the run, indicating foot arch structure suffered asymmetrical impacts throughout the run. It is plausible that the accumulated changes for each foot throughout the 10 KM was not the same. In one study (Fukano et al., 2021), foot arch symmetry was compared before and after a marathon and they reported significantly increased SI for the arch height ratio and no change in the navicular height drop after the run, indicating asymmetrical changes in foot arch shape. Another study reported inconsistent changes in foot posture index between left and right feet and no difference in the navicular height after the half marathon (Cowley and Marsden, 2013). Three factors may attribute to such inconsistency. Firstly, different subjects (recreational runners versus experienced runners or runners from a track and field club) were recruited. Accordingly, runners showed greater bilateral differences in foot morphology or metatarsal bones loading compared with the non-habitual exercisers (Maslon et al., 2017). Secondly, running distance (10 km versus half or full marathon) and road surfaces (synthetic rubber track vs. asphalt road) were different. Landing impact was largely associated with running road surfaces (Wang et al., 2012; Zhou et al., 2021) and the number of foot strikes is increased with running distance or duration, which would result in different load accumulation on foot. Thirdly, different measurements related to the foot arch structure were adopted. Cowley and Marsden, 2013 chose a semi-quantitative variable (foot posture index), which is also a subjective method; Fukano and co-authors (Fukano et al., 2021) only evaluated the shape of the foot arch. In our study, the foot morphology and foot arch were thoroughly assessed from the 3D foot model. A greater ground reaction force was found in the right foot than the left foot when the runners making a left turn during running and *vice versa*. This study was conducted on a standard 400-m synthetic rubber track and the runners ran in the same counterclockwise direction and made left turns for 24 times, which may lead to imbalance effects on right and left feet and finally observing the asymmetrical changes in foot arch after the run. Our findings demonstrated that running 10 km may be sufficient to increase foot asymmetry in the male recreational runners, particularly influencing the foot arch (the most important function structure of the foot), thereby may increase the risk of unilateral running-related injuries.

There are six limitations in this study. Firstly, all the data were collected in a static condition and the highlighted differences in foot morphology, foot arch and bilateral asymmetry were based on measurements from the 3D foot morphology before and after the 10 KM run. Due to lacking data under dynamic condition, this study cannot unveil the direct connection between the detected foot changes and the mechanisms of running-related injuries. Secondly, all data were collected based on one scan. Although the reliability was tested before data collection and the operator was an experienced technician, three scans would further improve reproducibility of the measurements. Thirdly, although running shoes were controlled through assigning the same model of running shoes, a comparative study would provide more information regarding the effects of running shoes. Although the participants were given enough time to find the right shoe size with the best perception of shoe-fit, perception of shoe-fit before and after the run was not quantified, and this study cannot gain insights into the effects of foot changes on shoe-fit. In addition, although this study investigated the acute effects following one running bout in their fastest pace and we have controlled other co-factors such as weightbearing physical activities (i.e., walking, running, jumping, etc.) within 24 h and age, we did not well controlled running experience during subject recruitment because feet tissue adaptation to running exercise may influence foot changes. Lastly, this study only recruited male recreational runners. Both male and female participants were recruited in previous studies (Cloughley and Mawdsley, 1995; Cowley and Marsden, 2013; Pan et al., 2023), however, the participants were not separated on gender during statistical analysis. Both genders should be recruited and analyzed separately to understand the interaction effects of gender and 10 KM run on foot morphology and foot arch.

## 5 Conclusion

Male recreational runners (minimal weekly running volume of 41.6 ± 24.8 km for 4.2 ± 4.4 years) barely altered their foot morphology but adjusted foot arch structure in both shape and stiffness after running 10 km at their fastest paces in a pair of traditional running shoes. They are prone to increasing stiffness of foot arch for adopting the compromised foot arch function (i.e., fatigued foot muscles, stretched plantar fascia, altered windlass mechanism) after the 10 km run. Meantime, 10 km would be sufficient to provoke asymmetrical changes of foot arch structure in the specific male recreational runners. Such alteration may demonstrate the necessary of wearing running shoes with foot arch support when they run 10 km or longer distance.

## Data Availability

The raw data supporting the conclusions of this article will be made available by the authors, without undue reservation.
