# Supplementary material for: Effects of 10 KM run on foot morphology and bilateral symmetry in male recreational runners
Source: Front Bioeng Biotechnol. 2024 Aug 13;12:1427418. doi: 10.3389/fbioe.2024.1427418 (PMC11347404; doi:10.3389/fbioe.2024.1427418)
Supplement: Supplementary file 1 [file Table1.DOCX]

Supplement Table 1 Intratester reliability and validity for different foot anthropometric parameters (Measurement #01 VS. Measurement #02).

| Parameters | Intraclass correlation coefficient (ICC) (mm) | Root mean squared error (RMSE) (mm) | Mean absolute error (MAE) (mm) | Percentage difference (%) |
| --- | --- | --- | --- | --- |
| Foot length | 0.999 | 0.6 | 0.4±0.5 | 0.2±0.2 |
| Truncated foot length | 0.981 | 2.5 | 1.9±1.7 | 1.0±0.9 |
| Ball width | 0.989 | 0.7 | 0.6±0.5 | 0.6±0.5 |
| Ball girth | 0.976 | 2.3 | 1.4±1.9 | 0.6±0.8 |
| Instep girth | 0.984 | 2.9 | 2.1±2.1 | 0.6±0.6 |
| Navicular height | 0.879 | 1.6 | 1.1±1.2 | 4.0±4.5 |
| Dorsum height | 0.972 | 1.2 | 0.7±1.0 | 1.1±1.6 |
| Hallux height | 0.929 | 1.0 | 0.6±0.8 | 2.2±3.0 |
